# Three-dimensional assessment of coronary high-intensity plaques with T1-weighted cardiovascular magnetic resonance imaging to predict periprocedural myocardial injury after elective percutaneous coronary intervention

**DOI:** 10.1186/s12968-019-0588-6

**Published:** 2020-01-16

**Authors:** Hayato Hosoda, Yasuhide Asaumi, Teruo Noguchi, Yoshiaki Morita, Yu Kataoka, Fumiyuki Otsuka, Kazuhiro Nakao, Masashi Fujino, Toshiyuki Nagai, Michikazu Nakai, Kunihiro Nishimura, Atsushi Kono, Yoshiaki Komori, Tomoya Hoshi, Akira Sato, Tomohiro Kawasaki, Chisato Izumi, Kengo Kusano, Tetsuya Fukuda, Satoshi Yasuda

**Affiliations:** 1 Department of Cardiovascular Medicine, National Cerebral and Cardiovascular Center, 6-1 Kishibe-Shimmachi, Suita, 564-8565 Japan; 2 Department of Advanced Cardiovascular Medicine, Graduate School of Medical Sciences, Kumamoto University, Kumamoto, Japan; 3 Department of Radiology, National Cerebral and Cardiovascular Center, Suita, Japan; 4 Department of Preventative Cardiology, National Cerebral and Cardiovascular Center, Suita, Japan; 5 Department of Research and Collaboration, Siemens Japan KK, Tokyo, Japan; 6 Department of Cardiovascular Medicine, University of Tsukuba, Tsukuba, Japan; 7 Cardiovascular Center, Shin-Koga Hospital, Kurume, Japan

**Keywords:** Magnetic resonance imaging, Coronary atherosclerosis, Percutaneous coronary intervention

## Abstract

**Background:**

Periprocedural myocardial injury (pMI) is a common complication of elective percutaneous coronary intervention (PCI) that reduces some of the beneficial effects of coronary revascularization and impacts the risk of cardiovascular events. We developed a 3-dimensional volumetric cardiovascular magnetic resonance (CMR) method to evaluate coronary high intensity plaques and investigated their association with pMI after elective PCI.

**Methods:**

Between October 2012 and October 2016, 141 patients with stable coronary artery disease underwent T1-weighted CMR imaging before PCI. A conventional 2-dimensional CMR plaque-to-myocardial signal intensity ratio (2D-PMR) and the newly developed 3-dimensional integral of PMR (3Di-PMR) were measured. 3Di-PMR was determined as the sum of PMRs above a threshold of > 1.0 for voxels in a target plaque. pMI was defined as high-sensitivity cardiac troponin T > 0.07 ng/mL.

**Results:**

pMI following PCI was observed in 46 patients (33%). 3Di-PMR was significantly higher in patients with pMI than those without pMI. The optimal 3Di-PMR cutoff value for predicting pMI was 51 PMR*mm^3^ and the area under the receiver operating characteristic curve (0.753) was significantly greater than that for 2D-PMR (0.683, *P* = 0.015). 3Di-PMR was positively correlated with lipid volume (r = 0.449, *P* < 0.001) based on intravascular ultrasound.

Stepwise multivariable analysis showed that 3Di-PMR ≥ 51 PMR*mm^3^ and the presence of a side branch at the PCI target lesion site were significant predictors of pMI (odds ratio [OR], 11.9; 95% confidence interval [CI], 4.6–30.4, *P* < 0.001; and OR, 4.14; 95% CI, 1.6–11.1, *P* = 0.005, respectively).

**Conclusions:**

3Di-PMR coronary assessment facilitates risk stratification for pMI after elective PCI.

**Trial registration:**

retrospectively registered.

## Introduction

Percutaneous coronary intervention (PCI) has become the most common procedure for coronary revascularization in patients with both stable and unstable coronary artery disease (CAD). Although technical advances in PCI have resulted in a safer therapeutic procedure, 5–30% of patients undergoing elective PCI demonstrate evidence of periprocedural myocardial injury (pMI) from the procedure itself [1, 2]. The extent of pMI as determined by evaluation of creatine phosphokinase MB isoenzyme, high-sensitivity troponin T (hs-cTnT), and troponin I is significant enough to be prognostically important [1, 3]. Therefore, pMI might reduce some of the beneficial effects of coronary revascularization.

Coronary high-intensity plaques (HIPs) with a high plaque-to-myocardial signal intensity ratio (PMR) on non-contrast T1-weighted (T1w) cardiovascular magnetic resonance (CMR) imaging are associated with future coronary events and PCI-related pMI [4–7]. However, current HIP-PMR evaluation is solely based on signal intensity without consideration of plaque volume, which yields a 2-dimensional PMR (2D-PMR) based on a coronary plaque as a region of interest (ROI) [4, 5]. Since larger plaque volume is associated with cardiovascular events such as acute myocardial infarction, which includes pMI [8, 9], more accurate, quantitative assessment is needed to predict cardiovascular events.

Herein, we sought to examine whether 3-dimensional integral (3Di) volumetric assessment of coronary HIPs on non-contrast T1w imaging would have better predictive value for pMI after elective PCI compared with the current 2D-PMR in patients with stable CAD.

## Methods

### Study patients

From October 2012 to October 2016, 215 consecutive stable CAD patients undergoing elective PCI following non-contrast T1w imaging for coronary atherosclerosis were included in this study retrospectively. CMR was used to evaluate the characteristics of the target coronary plaque. We excluded 74 patients who had lesions involving the left main trunk (*n* = 12), chronic total occlusion (*n* = 17), previous cardiac surgery (n = 1), renal insufficiency (serum creatinine ≥1.8 mg/dL, n = 1), multi-segment PCI (*n* = 2), missing baseline hs-cTnT data (*n* = 21), poor CMR image quality (*n* = 8), or rotational or direct coronary atherectomy during PCI (n = 12). Ultimately, 141 patients (122 males, 87%) with a median age of 68 years (interquartile range [IQR], 60–73 years) were analyzed in this study. This study was approved by our institutional review board (M26–037-2).

### CMR coronary plaque imaging

Non-contrast T1w imaging was performed at 3 T (MAGNETOM Verio; Siemens AG Healthcare Sector, Erlangen, Germany) with a 32-channel cardiac coil. The procedures used to acquire CMR images in this study have been previously described [6, 10, 11]. Briefly, coronary plaque imaging was performed using an inversion recovery-prepared 3D T1w turbo fast low-angle shot sequence with an electrocardiographic (ECG) trigger, navigator-gated free-breathing, and fat suppression. Transaxial sections covered the entire heart (inversion time, 650 ms; field of view, 280 mm × 228 mm; acquisition matrix, 256 × 187; reconstruction matrix, 512 × 374; acquisition slice thickness, 1.0 mm; reconstruction spatial resolution, 0.55 mm × 0.60 mm × 1.0 mm; repetition time/echo, 4.7 ms/2.13 ms; flip angle, 12°; GRAPPA factor, 2; navigator gating window, ±1.5–2.5 mm; and data acquisition window, 84–120 ms). The average navigator efficiency was 35.7 ± 8.2%. To identify the coronary segments of plaques evaluated on T1w images in patients who did not undergo computed tomography angiography (CTA), 3D coronary CMR angiograms using a free-breathing, navigator-gated, ECG-triggered, fat-saturated, segmented gradient echo sequences were obtained in the axial plane to cover the entire heart (repetition time, 3.27 ms; echo time, 1.35 ms; flip angle, 12°; field of view, 280 × 227; acquisition matrix, 256 × 175; reconstruction matrix, 512 × 350, acquisition slice thickness 1.0 mm; acquisition special resolution, 1.30 mm × 1.09 mm × 1.56 mm; reconstruction spatial resolution, 0.55 mm × 0.60 mm × 1.0 mm; partial Fourier, 6/8; data window duration, 98 ms). Trigger delay and acquisition windows were based on the duration of minimal right coronary artery motion as determined on cine-CMR imaging. The mean acquisition times for plaque imaging and coronary CMR angiography were 23.1 ± 3.8 min and 17.4 ± 2.4 min, respectively.

### Three-dimensional plaque analysis with CMR

On CMR analysis, the coronary vasculature tree was subdivided into 8 segments [12]. In brief, the right coronary artery was analyzed in 3 segments (segments 1, 2, 3). The left coronary artery was analyzed in 4 segments that comprised the left anterior descending artery (segments 6, 7) and left circumflex artery (segments 11, 13). Since lesions in the left main trunk, both in the left anterior descending artery and left circumflex artery (segment 5), are associated with a large ischemic burden, PCI for left main stenosis is indicated only in patients with a low SYNTAX score and non-multivessel disease [13], and PCI for left main lesions may be susceptible to operator bias. Therefore we excluded left main lesions in the present analysis. For segment identification, segments were pre-defined according to the distance from the vessel’s origin [5, 6, 12]. The location of a coronary plaque was determined by carefully comparing either CTA or CMR angiogram or invasive coronary arteriography images during PCI using fiduciary points (e.g. side branches and vessel bends). Once a coronary plaque had been confirmed with either CTA or coronary CMR angiogram, the corresponding areas on coronary T1w images were carefully matched using the surrounding cardiac and chest wall structures [11]. Fifty-seven patients underwent coronary CTA and co-registration between coronary T1w imaging and CTA (Additional file 1: Supplemental Methods and Table S1).

**Fig. 1:**
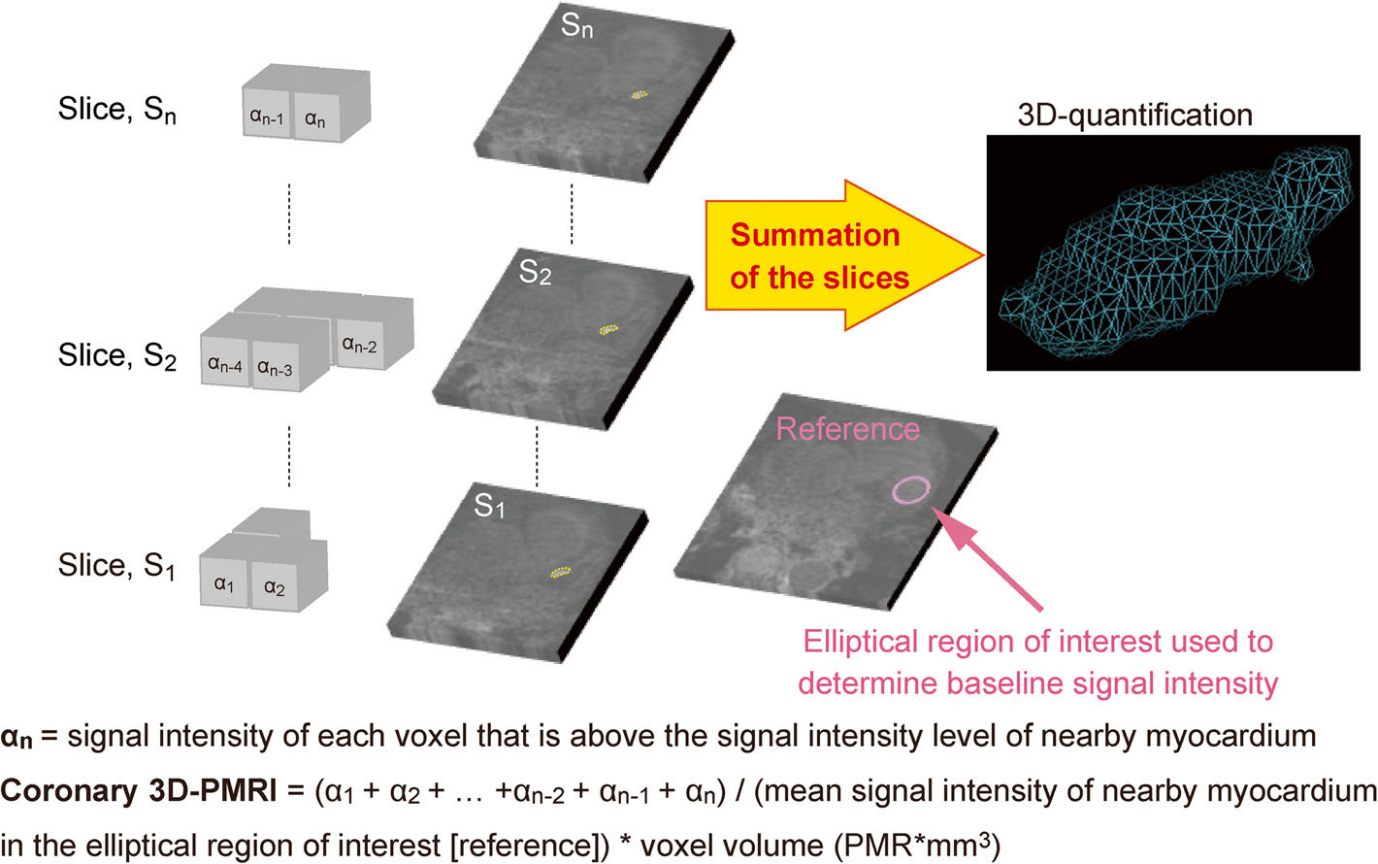
Principle behind 3-dimensional (3D) plaque assessment on T1-weighted imaging. Gray cubes represent voxels. α_n_ represents the signal intensity of each voxel with higher signal intensity than that of nearby myocardium. Entire voxels of a coronary plaque that are above the signal intensity of nearby myocardium (plaque-to-myocardial signal intensity ratio > 1.0) were segmented within contiguous slices (surrounded by yellow dotted lines) to calculate the integral of signal intensity and voxel volume

**Fig. 2:**
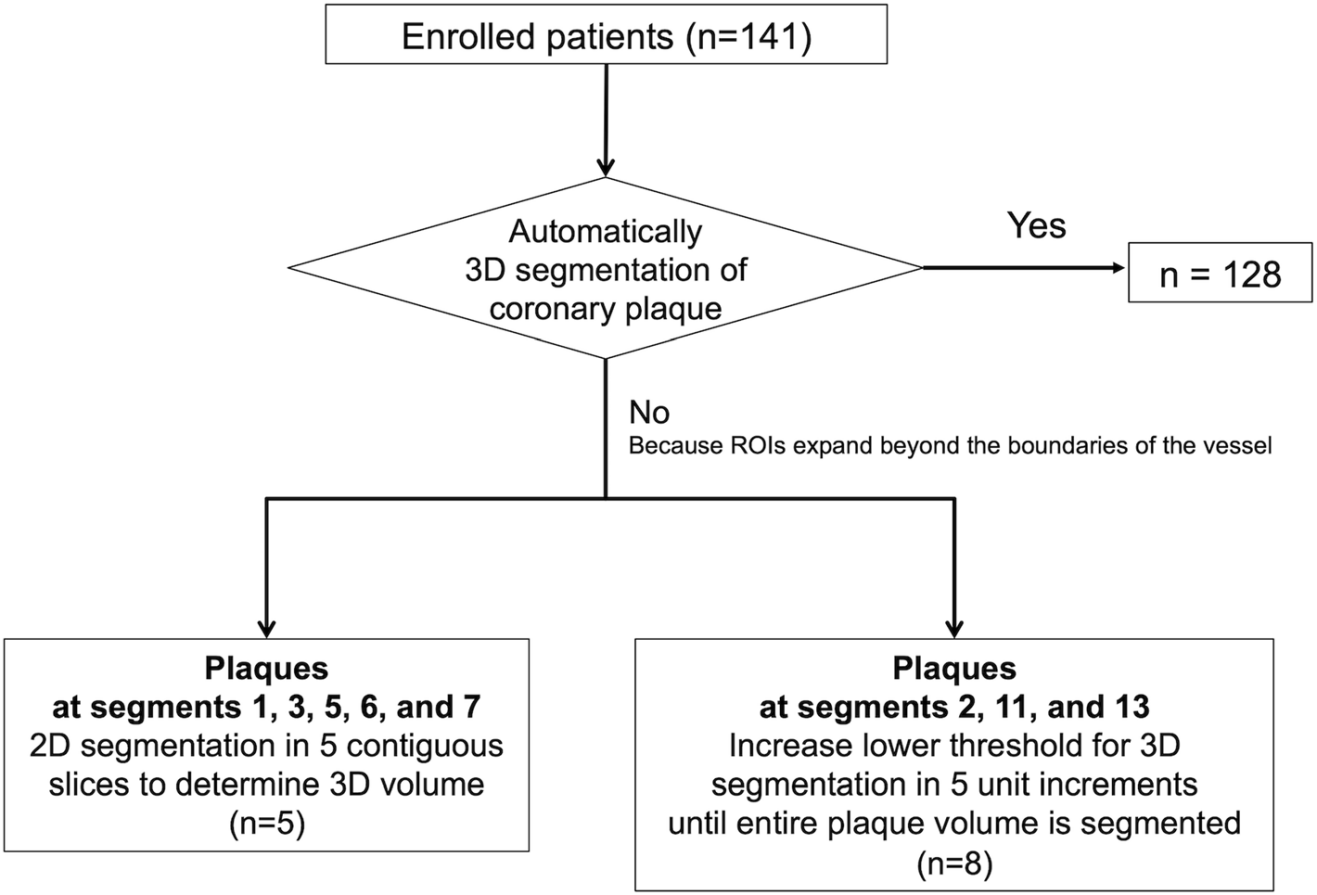
Flow chart for 3D plaque assessment on T1-weighted imaging

2D-PMR was assessed as previously described [4–6, 11]. For 3D analysis of coronary plaques on non-contrast T1w imaging, OsiriX MD software (version 8.0.2, Pixmeo, Geneva, Switzerland) was used. The 3D integral of the plaque-to-myocardium signal intensity ratio (3Di-PMR) was defined as the integral of voxel volume (0.55 mm × 0.60 mm × 1.0 mm = 0.33 mm^3^) multiplied by its PMR value > 1.0 from a coronary plaque (Fig. 1, Additional file 1: Figure S1, Figure S2, Figure S3, and Additional file 2: Step 1, Videos S1, Additional file 3: Step 2, Videos S2, Additional file 4: Step 3, Videos S3). The algorithm for plaque segmentation was based on a region-growing technique, a function built into the OsiriX MD software consisting of the following steps (Figure 2 and Additional file 2: Step 1, Videos S1, Additional file 3: Step 2, Videos S2, Additional file 4: Step 3, Videos S3) [14]. First, an elliptical ROI of ≥1.0 cm^2^ in area was drawn around the myocardium near a coronary plaque. The mean signal intensity of the ROI was defined as the lower threshold of the signal intensity of the coronary plaque. Next, entire voxels of a coronary plaque above the threshold PMR of 1.0 were segmented automatically within contiguous slices to calculate both the integral signal intensity and voxel volume (3D region growing: Step1, Additional file 1: Figure S1 and Additional file 2: Step 1, Video S1) [14]. Segments with a PMR above the threshold that were not analyzed by the automatic 3D segmentation, which was defined as those with expansion of the ROI beyond the boundaries of the vessel, were divided into 2 groups according to the course of the coronary vessel: those running along axial slices (Step 2, segments 1, 3, 6, 7) and those running perpendicular to axial slices (Step 3, segments 2, 11, 13). These segments were then analyzed using the methods described below.

Since the mean diameter of proximal coronary segments in Japanese subjects is 4–5 mm [15], we used a slice thickness of 1 mm to cover the entirety of each vessel. Therefore, in the Step 2 method (for segments 1, 3, 6, 7), 3Di-PMR was determined by performing automatic contiguous 2D segmentation of each coronary plaque above the PMR threshold, in not more than 5 contiguous slices and without expansion beyond the boundaries of the vessel (Additional file 1: Figure S2-a, S2-b and Additional file 3: Step 2, Video S2).

For segments 2, 11, 13, the lower segmentation threshold was increased from the signal intensity of the nearby myocardium in increments of 5 units until there was no longer expansion beyond the vessel boundaries (Additional file 1: Figure S3 and Additional file 4: Step 3, Video 3).

### PCI

PCI was performed as previously reported [16]. pMI after elective PCI was defined as an increase in serum hs-cTnT levels to more than 5 times the upper limit of normal (0.07 ng/mL) at 24 h after PCI. Slow flow was defined as the Thrombolysis In Myocardial Infarction (TIMI) grade 0, 1, or 2 flow in the distal infarct-related artery despite the absence of occlusion or dissection at the treatment site. The presence of a side branch was defined as the presence of a vessel of ≥1.5 mm in diameter within the target lesion. Side branch occlusion was defined as TIMI grade 0, 1, or 2 flow during PCI.

### Intravascular ultrasound image analysis

Intravascular ultrasound (IVUS) images were obtained during PCI and analyzed in 126 patients. IVUS was performed using a commercially available IVUS catheter (View It; Terumo, Tokyo, Japan) with 0.5 mm/sec auto-pullback. Quantitative and qualitative analysis were performed in a blinded manner as previously described [4, 6, 7]. Positive remodeling was defined as a remodeling index of > 1.05. Ultrasound attenuation was defined as IVUS images with backward signal attenuation of ≥180° behind the plaque without dense calcium. The presence of ultrasound attenuation was defined as longitudinal attenuation length of ≥5 mm [17]. For tissue characterization, IVUS data were analyzed using the manufacturer’s default setting on the basis of previous data used to define a range of integrated backscatter values. Coronary plaques were classified into three categories: lipid pool (blue), fibrosis (green/yellow), and calcification (red) [9].

### Statistical analysis

Values are presented as medians (IQR). Values were compared using the Mann-Whitney U test or Kruskal-Wallis test for multiple comparisons followed by the Steel-Dwass test for post hoc analysis. Categorical baseline variables were compared using Fisher’s exact test or the chi-squared test as appropriate. 3Di-PMR and 2D-PMR cutoff values for the development of coronary events were determined with receiver operating characteristic (ROC) analysis. To identify risk factors for pMI, univariable and multivariable logistic regression models were constructed using 3Di-PMR, attenuation, positive remodeling, calcification, type B2/C lesion, and presence of a side branch. Stepwise multivariable logistic regression with a *P* value of 0.10 for backward elimination was performed to select the best predictive model. All analyses were conducted using JMP (version 12, SAS Japan, Tokyo, Japan) and Stata, version 14 (StataCorp LP, College Station, Texas, USA). A *P* value less than 0.05 was considered statistically significant.

## Results

Table 1 summarizes the baseline characteristics of the study patients. Of 141 patients, pMI was observed in 46 patients (33%). Both 3Di-PMR and 2D-PMR were significantly higher in patients with pMI than those without pMI (*P* < 0.001), although age, gender, coronary risk factors, medications, and pre-PCI hs-cTnT level were similar. As shown in Additional file 1: Figure S4-C, 3Di-PMR is strongly and positively correlated with 2D-PMR (r = 0.832, *P* < 0.001). Intraclass correlation coefficients with 95% confidence intervals (CIs) were calculated to assess intra- and inter-reader agreement regarding 3Di-PMR. The interval between initial analysis of 3Di-PMR of HIPs and secondary analysis was 3 months. The intra- and inter-reader intraclass correlation coefficients for 3Di-PMR were 0.947 (95% CI, 0.911–0.969) and 0.926 (95% CI, 0.876–0.957), respectively. All correlation coefficients for calculating 3Di-PMR were greater than 0.8, indicating good intra- and inter-observer agreement.

**Table 1:** Baseline Characteristics of the Study Patients

	No pMI (*n* = 95)	pMI (*n* = 46)	*P* Value
Age, yrs	68 (61–74)	67 (59–73)	0.582
Male	83 (87)	39 (85)	0.793
BMI, kg/m^2^	25 (22–27)	24 (22–27)	0.514
Hypertension	76 (80)	35 (76)	0.662
Hyperlipidemia	86 (91)	42 (91)	1.000
Diabetes mellitus	29 (31)	12 (26)	0.627
Current smoker	11 (12)	7 (15)	0.594
Total cholesterol, mg/dL	149 (134–173)	160 (144–177)	0.338
LDL cholesterol, mg/dL	82 (67–102)	82 (71–100)	0.811
HDL cholesterol, mg/dL	44 (38–52)	46 (38–55)	0.273
Triglycerides, mg/dL	135 (97–192)	129 (94–167)	0.356
Hemoglobin A_1c_, %	6.0 (5.6–6.3)	5.9 (5.7–6.2)	0.378
Serum creatinine, mg/dL	0.90 (0.79–1.05)	0.93 (0.81–1.06)	0.633
Multivessel disease	28 (29)	18 (39)	0.258
Previous MI	14 (15)	10 (22)	0.342
LVEF, %	60 (55–65)	60 (57–65)	0.732
hs-cTnT before PCI, ng/mL	0.008 (0.006–0.012)	0.009 (0.006–0.016)	0.543
hs-cTnT after PCI, ng/mL	0.027 (0.020–0.042)	0.138 (0.094–0.276)	< 0.001
Medications			
Statin	88 (93)	41 (89)	0.527
Beta-blocker	67 (71)	27 (59)	0.185
ACE inhibitor or ARB	50 (53)	24 (52)	1.000
MRI findings			
2D-PMR	1.15 (0.98–1.36)	1.66 (1.09–2.93)	< 0.001
3Di-PMR, PMR*mm^3^	9 (0–49.8)	88.4 (32.2–203.7)	< 0.001
Angiographic findings and PCI parameters			
Mean reference vessel diameter (mm)	3.50 (3.06–4.19)	3.45 (3.07–4.24)	0.852
Ratio of the maximal balloon diameter to the vessel diameter	0.95 (0.79–1.06)	0.96 (0.83–1.06)	0.852
Total lesion length (mm)	16 (10–26)	18 (12–28)	0.439

Values are medians (interquartile range) or n (%)

*2D-PMR* 2-dimensional plaque-to-myocardium signal intensity ratio, *3Di-PMR* 3-dimensional integral of the plaque-to-myocardium signal intensity ratio, *ACE* Angiotensin-converting enzyme, *ARB* Angiotensin II receptor blocker, *BMI* Body mass index, *HDL* High-density lipoprotein, *hs-cTnT* High-sensitivity cardiac troponin T, *LDL* Low-density lipoprotein, *LVEF* Left ventricular ejection fraction, *MI* Myocardial infarction, *MRI* Magnetic resonance imaging, *PCI* Percutaneous coronary intervention, *pMI* Periprocedural myocardial injury, PMR*mm^3^ the unit of 3Di-PMR; defined as the integral of voxel volume multiplied by its PMR value > 1.0 from a coronary plaque

**Fig. 3:**
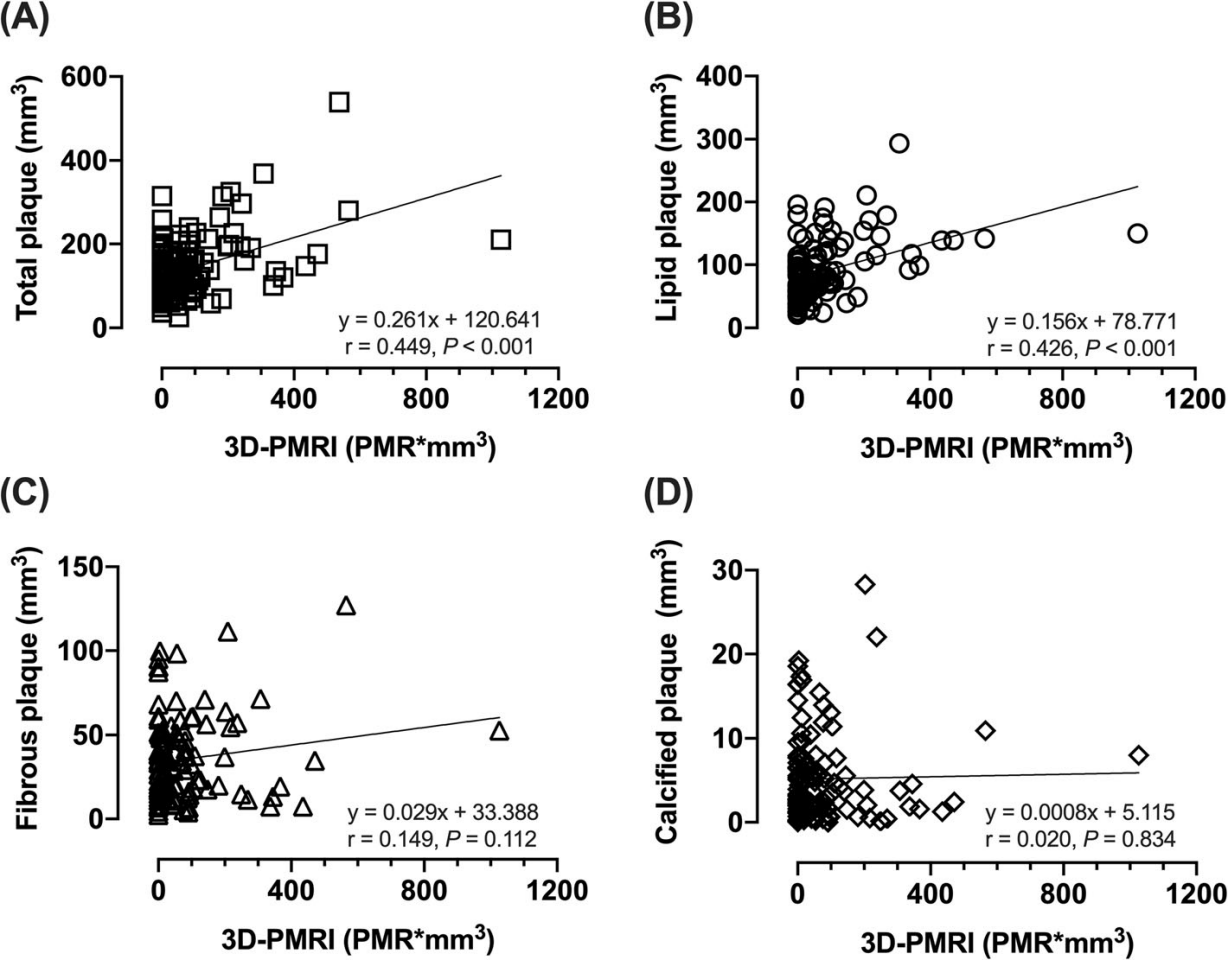
Correlation between 3D integral (3Di)-plaque to myocardial signal intensity ratio (PMR) and plaque characteristics based on integrated backscatter intravascular ultrasound. Correlation between 3Di-PMR and total plaque volume (**a**), lipid plaque volume (**b**), fibrous plaque volume (**c**), and calcified plaque volume (**d**) are shown

On the basis of the ROC analysis, a 3Di-PMR value of 51 PMR*mm^3^ was identified as the best cutoff for predicting pMI after elective PCI, with an area under the curve (AUC) of 0.753 (95% CI, 0.665–0.841). At this value, the sensitivity and specificity for predicting pMI were 74 and 76%, respectively. Table 2 and Additional file 1: Figure S4-D and S4-E show the lesion characteristics and incidence of pMI and slow flow phenomenon by 3Di-PMR cutoff value. Patients with 3Di-PMR ≥ 51 PMR*mm^3^ had a larger plaque volume, longer attenuation length, higher remodeling index, larger lipid volume, and higher incidence of both pMI and slow flow phenomenon than those with 3Di-PMR < 51 PMR*mm^3^. To show the clinical significance of 3Di-PMR, previously described predictors of pMI were compared with 3Di-PMR. From ROC analysis (Table 3), 3Di-PMR had a significantly higher AUC (0.753) than 2D-PMR (0.683 [95%CI, 0.609–0.782]) on T1w imaging (*P* = 0.015), as well as other IVUS or coronary angiography derived indices; attenuation length (0.641 [95% CI, 0.539–0.477], *P* = 0.038), remodeling index (0.547 [95% CI, 0.456–0.637], *P* = 0.013), and prevalence of type B2/C lesion (0.525 [95% CI, 0.438–0.613], *P* < 0.001), bifurcation lesion (0.612 [95% CI, 0.532–0.692], *P* = 0.039), and calcification (0.503 [95% CI, 0.436–0.570], *P* < 0.001). Figure 3 shows the correlation between 3Di-PMR and IVUS derived indices. 3Di-PMR was significantly positively correlated with total plaque volume and lipid volume (r = 0.449, *P* < 0.001 and r = 0.426, *P* < 0.001, respectively).

**Table 2:** Lesion Characteristics Stratified by 3Di-PMR Cutoff

	3Di-PMR < 51 PMR*mm^3^(*n* = 84)	3Di-PMR ≥51 PMR*mm^3^(*n* = 57)	*P* Value
Angiographic findings			
Target vessel			0.375
LAD/LCX/RCA	52/14/18	29/9/19	
Type B2/C lesion	46 (55)	35 (61)	0.434
Side branch at the PCI target lesion site	53 (63)	33 (58)	0.534
Calcification	17 (20)	7 (12)	0.217
Total lesion length, mm	17 (10–26)	18 (11–28)	0.769
PCI parameters			
Stent diameter, mm	3.0 (2.8–3.3)	3.25 (3.0–3.5)	< 0.001
Stent length, mm	24 (18–33)	24 (18–37)	0.500
Post-dilatation balloon size, mm	3.3 (3.0–3.5)	3.5 (3.3–4.0)	< 0.001
Cross-sectional IVUS parameters	(n = 74)	(n = 52)	
Grayscale IVUS analysis			
Lesion EEM CSA, mm^2^	8.4 (6.1–11.3)	12.9 (8.9–15.8)	< 0.001
Lesion lumen CSA, mm^2^	1.3 (1.0–2.0)	1.3 (0.9–2.0)	0.560
Lesion P + M CSA, mm^2^	6.6 (5.0–9.4)	11.3 (8.1–13.5)	< 0.001
Plaque burden, %	83 (77–86)	89 (85–92)	< 0.001
Remodeling index	0.89 (0.78–1.05)	1.06 (0.91–1.19)	< 0.001
Positive remodeling	19 (26)	26 (50)	0.004
Ultrasound attenuation	6 (8)	18 (35)	< 0.001
Attenuation length, mm	0 (0–1.7)	3.6 (0–6.6)	< 0.001
Intracoronary thrombus	2 (3)	9 (18)	0.015
IB-IVUS parameters			
Lipid area, %	68 (57–80)	81 (68–86)	0.003
Fibrous area, %	24 (17–38)	17 (13–28)	0.007
Calcified area, %	3.7 (1.1–7.4)	2.0 (0.4–3.7)	0.030
Volumetric IVUS analysis			
Grayscale IVUS parameters			
EEM volume, mm^3^	174 (135–247)	246 (171–336)	< 0.001
Lumen volume, mm^3^	62 (47–93)	75 (57–107)	0.147
Total plaque volume, mm^3^	104 (82–140)	158 (114–210)	< 0.001
Plaque burden, %	61 (55–67)	66 (62–74)	< 0.001
IB-IVUS parameters			
Lipid volume, %	68 (55–78)	71 (63–83)	0.039
Fibrous volume, %	28 (20–40)	25 (16–33)	0.052
Calcified volume, %	3.5 (1.6–7.3)	2.4 (0.9–4.0)	0.039

Values are medians (interquartile range) or n (%)

*2D-PMR* 2-dimensional plaque-to-myocardium signal intensity ratio, *3Di-PMR* 3-dimensional integral of the plaque-to-myocardium signal intensity ratio, *CSA* Cross-sectional area, *EEM* External elastic membrane, *IB-IVUS* Integrated backscatter intravascular ultrasound, *LAD* Left anterior descending coronary artery, *LCX* Left circumflex coronary artery, *P + M* Plaque plus media, *PCI* Percutaneous coronary intervention, PMR*mm^*3*^ the unit of 3Di-PMR; the integral of voxel volume multiplied by its PMR value > 1.0 from a coronary plaque, *RCA* Right coronary artery

**Table 3:** Receiver Operating Characteristic Analysis Demonstrating the Prediction of Periprocedural Myocardial Injury

Variable	AUC (95% CI)	*P* Value
3Di-PMR, PMR*mm^3^	0.753 (0.665–0.841)	Referent
2D-PMR	0.683 (0.609–0.782)	0.015
Attenuation length	0.641 (0.539–0.744)	0.038
Remodeling index	0.547 (0.456–0.637)	0.013
Side branch	0.612 (0.532–0.692)	0.039
Type B2/C lesion	0.525 (0.438–0.613)	< 0.001
Calcification	0.503 (0.436–0.570)	< 0.001

*3Di-PMR* 3-dimensional integral of the plaque-to-myocardium signal intensity ratio, *AUC* Area under the curve, *CI* Confidence interval, PMR*mm^*3*^ the unit of 3Di-PMR; the integral of voxel volume mulplied by its PMR value > 1.0 from a coronary artery

**Fig. 4:**
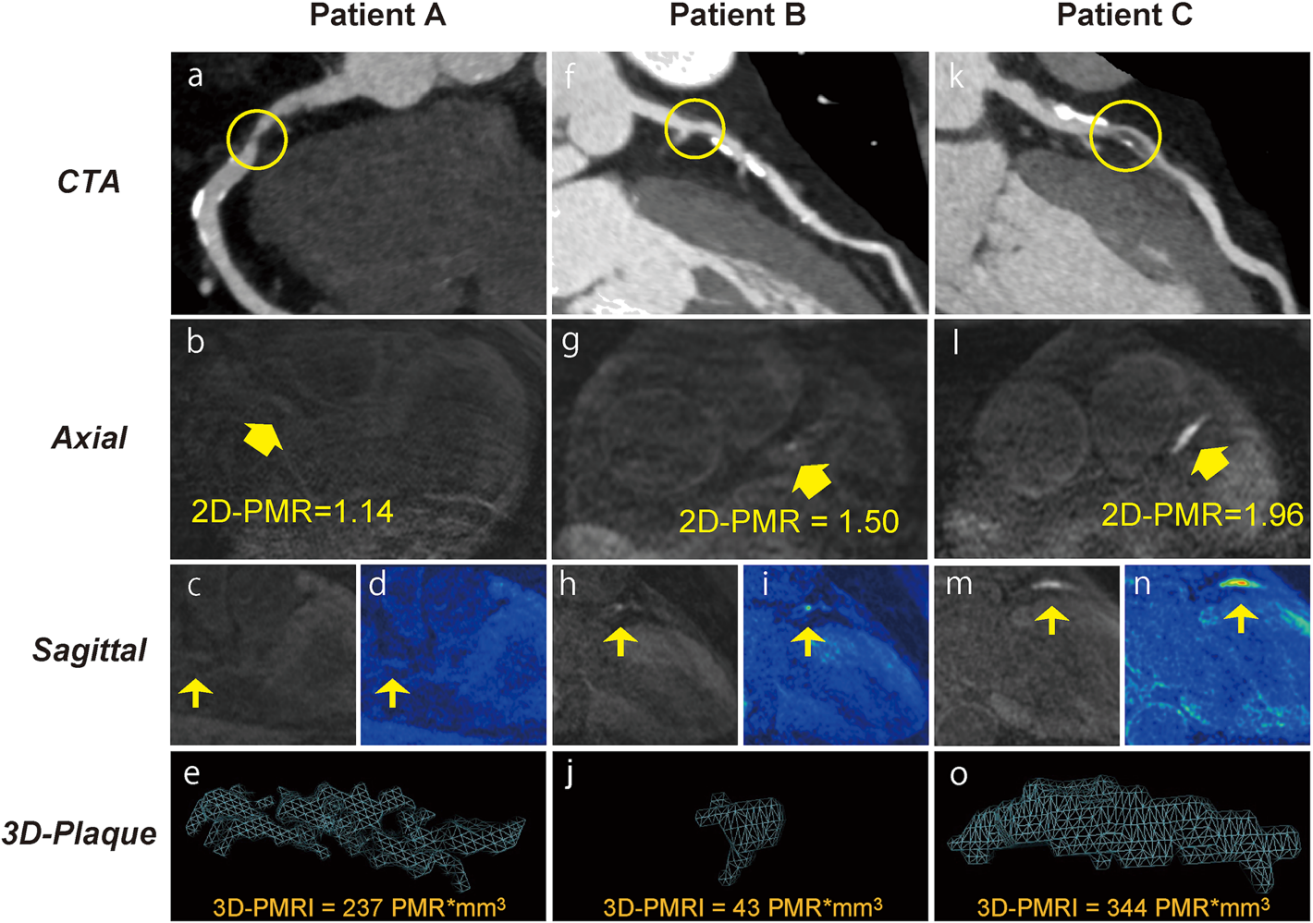
Representative 2-dimensional and 3-dimensional plaque assessment on T1-weighted imaging. Coronary plaques with 2D^low^3D^high^ in the proximal right coronary artery (2D-PMR, 1.14; 3Di-PMR, 237 PMR*mm^3^; Patient A: a–e), 2D^high^3D^low^ in the proximal left anterior descending artery (LAD) (2D-PMR, 1.50; 3Di-PMR, 43 PMR*mm^3^; Patient B: f–j), and 2D^high^3D^high^ in the proximal LAD (2D-PMR, 1.96; 3Di-PMR, 344 PMR*mm^3^; Patient C: k–o). Computed tomography angiography (CTA) images (a, f, k), and axial images (b, g, l), sagittal images (c, h, m), color maps (d, I, n), and 3D region of interests (3D plaque: e, j, n) on T1w images are shown. Yellow circles indicate percutaneous coronary intervention target lesion sites on CTA. Yellow arrows indicate lesions on T1w imaging corresponding to a lesion on angiography that underwent intervention.

**Fig. 5:**
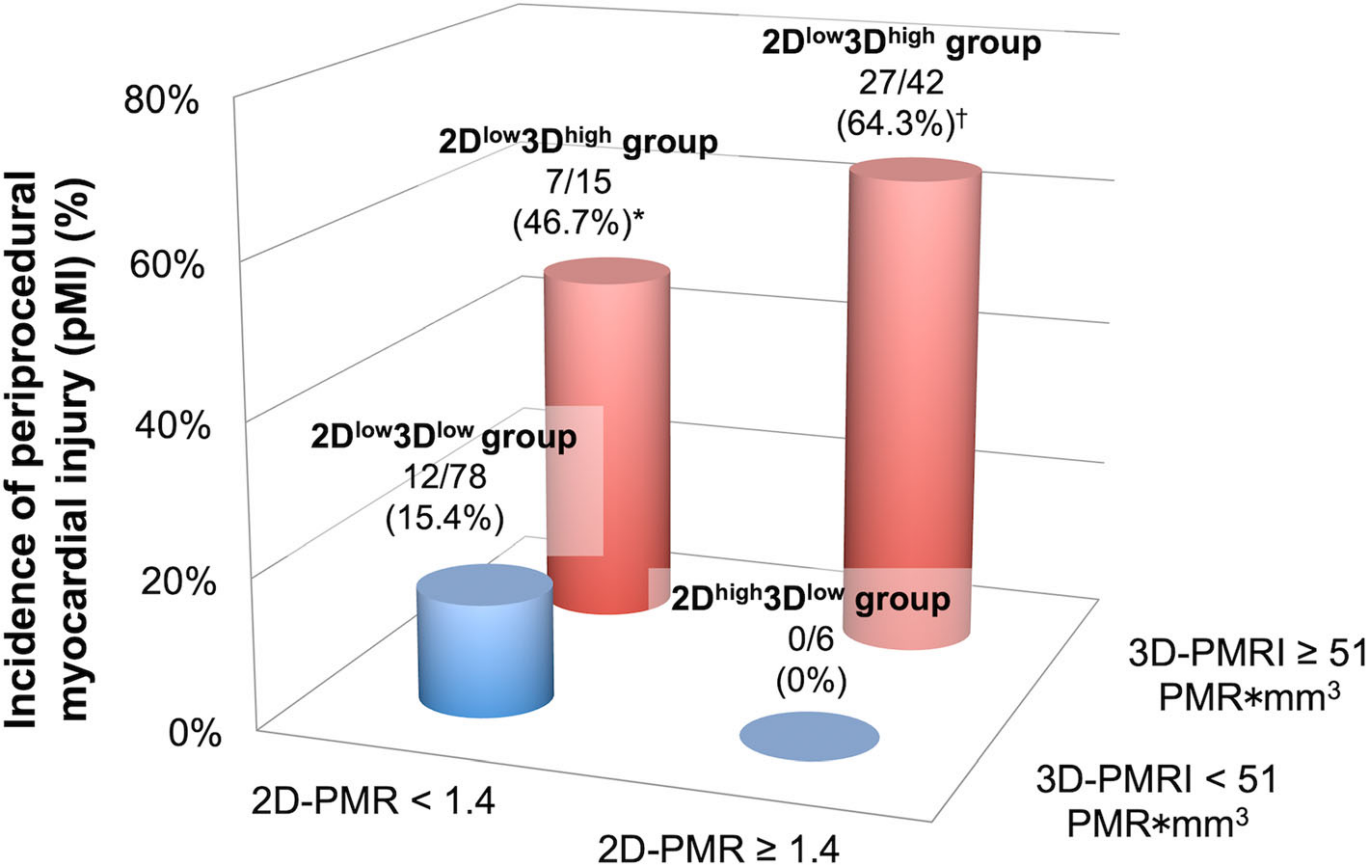
Incidence of periprocedural myocardial injury (pMI) based on 3Di-PMR and 2D-PMR cutoff values. The red and blue bars represent patients with 3Di-PMR ≥ 51 PMR*mm^3^ and < 51 PMR*mm^3^, respectively. *P* < 0.001 based on the chi-squared test. * *P* = 0.006 vs. 2D^high^3D^low^ group. ^†^*P* < 0.001 vs. 2D^low^3D^low^ group, and *P* = 0.003 vs. 2D^high^3D^low^ group

Because our previous study had demonstrated that a 2D-PMR cutoff value of 1.4 is a significant predictor of coronary events [5], we subdivided the study patients into the following 4 groups according to the 3Di-PMR cutoff value of 51 PMR*mm^3^ and the 2D-PMR cutoff value of 1.4: 2D-PMR < 1.4 + 3Di-PMR < 51 PMR*mm^3^ (2D^low^3D^low^ group: *n* = 78), 2D-PMR ≥ 1.4 + 3Di-PMR < 51 PMR*mm^3^ (2D^high^3D^low^ group: *n* = 6), 2D-PMR < 1.4 + 3Di-PMR ≥ 51 PMR*mm^3^ (2D^low^3D^high^ group: *n* = 15), and 2D-PMR ≥ 1.4 + 3Di-PMR ≥ 51 PMR*mm^3^ (2D^high^3D^high^ group: *n* = 42). Figure 4 shows representative 2D and 3D plaque assessments on T1w imaging. Coronary plaques with 2D-PMR < 1.4 + 3Di-PMR ≥ 51 PMR*mm^3^ in the proximal right coronary artery (a patient with 2D^low^3D^high^ plaque; Patient A: panels a–e), 2D-PMR ≥ 1.4 + 3Di-PMR < 51 PMR*mm^3^ in the proximal left anterior descending artery (a patient with 2D^high^3D^low^ plaque; Patient B: panels f–j), and 2D-PMR ≥ 1.4 + 3Di-PMR ≥ 51 PMR*mm^3^ in the proximal left anterior descending artery (a patient with 2D^high^3D^high^ plaque; Patient C: panels k–o) and corresponding CTA images are shown. Coronary CTA (a, f, k), and axial images (b, g, l), sagittal images (c, h, m), color maps (d, I, n), and volume images (3D plaque: e, j, n) and non-contrast T1w images are shown in Figure 4. Among the four groups based on 2D-PMR and 3Di-PMR cutoff values, the incidence of pMI was highest in the 2D^high^3D^high^ group (64.3%), and lowest in the 2D^high^3D^low^ group (0%) (*P* < 0.001, Figure 5). Of note, even in patients with 2D-PMR < 1.4, the incidence of pMI among those with 3Di-PMR ≥ 51 PMR*mm^3^ was significantly higher than in patients with 3Di-PMR < 51 PMR*mm^3^ (2D^low^3D^high^ group: 46.7% vs. 2D^low^3D^low^ group: 15.4%, *P* = 0.006; Figure 5).

As shown in Table 4, univariable Cox regression analysis showed that 3Di-PMR ≥ 51 PMR*mm^3^, attenuation detected on IVUS, and the presence of a side branch at the PCI target lesion site were associated with pMI. Multivariable analysis showed that 3Di-PMR ≥ 51 PMR*mm^3^ and the presence of a side branch at the PCI target lesion site were significant predictors of pMI (odds ratio [OR], 11.9; 95% CI, 4.63–30.4; *P* < 0.001 and OR, 4.14; 95% CI, 1.55–11.1, *P* = 0.005; respectively).

**Table 4:** Univariable and Multivariable Analyses of Predictors for Periprocedural Myocardial Injury

Variable	Univariable			Multivariable			Stepwise		
	OR	95% CI	*P*	OR	95% CI	*P*	OR	95% CI	*P*
3Di-PMR ≥51 PMR*mm^3^	8.87	(3.95–19.9)	< 0.001	10.7	(3.85–29.7)	< 0.001	11.9	(4.63–30.4)	< 0.001
Ultrasound attenuation	3.52	(1.41–8.83)	0.007	2.71	(0.83–8.87)	0.100			
Calcification	1.04	(0.41–2.64)	0.935	0.86	(0.24–3.08)	0.818			
Type B2/C lesion	1.23	(0.60–2.53)	0.568	0.57	(0.22–1.53)	0.266			
Positive remodeling	1.49	(0.70–3.19)	0.301	0.98	(0.37–2.63)	0.969			
Side branch	2.75	(1.25–6.04)	0.012	5.83	(1.86–18.3)	0.003	4.14	(1.55–11.1)	0.005
Total plaque volume (per 1-mm^3^ increase)	1.00	(1.00–1.01)	0.170	1.00	(0.99–1.01)	0.879			

*3Di-PMR* 3-dimensional integral of the plaque-to-myocardium signal intensity ratio, *CI* Confidence interval, *OR* Odds ratio, PMR*mm^*3*^ the unit of 3Di-PMR; defined as the integral of voxel volume multiplied by its PMR value > 1.0 from a coronary artery

Finally, we compared between the 3Di-PMR and coronary CTA derived predictors of pMI previously described to be indicative of high-risk plaque among the 57 patients who underwent CTA (Additional file 1: Tables S1–S2). From ROC analysis, 3Di-PMR also had higher AUC (0.777 [95% CI, 0.644–0.910]) than CTA-derived indices with significance or marginal significance; CT value (0.609, [95% CI, 0.644–0.910]; *P* = 0.051), remodeling index (0.618, [95% CI, 0.461–0.774]; *P* = 0.051), low attenuation plaque (LAP; 0.566 [95%CI, 0.427–0.704], *P* = 0.007), positive remodeling (PR; 0.579 [95% CI, 0.445–0.713], *P* = 0.007), spotty calcification (0.526 [95% CI, 0.367–0.666], *P* = 0.022), and LAP + PR (0.651 [95% CI, 0.501–0.801], *P* = 0.068).

## Discussion

The major finding of this study is that the presence of a coronary HIP with 3Di-PMR ≥ 51 PMR*mm^3^ detected by non-contrast T1w imaging was a significant independent predictor of PCI-related pMI compared to conventional 2D-PMR and other IVUS- or CAG-derived predictors. Thus, noninvasive characterization of coronary plaques based on 3Di-PMR with CMR, which includes the concept of plaque burden extent, is clinically informative for risk stratification of patients with elective PCI.

Although PCI is an important coronary revascularization strategy, especially in patients with CAD, the effect of PCI on clinical outcomes is limited despite the development of contemporary PCI techniques and devices [18]. Thus, reducing the number of complications during PCI may improve the clinical outcomes of PCI. pMI is a complication of PCI whose mechanism is thought to be related to side branch occlusion, atherosclerotic embolism, or both during PCI and may be associated with cardiovascular events after PCI [2, 3]. The present study shows that novel 3D evaluation of coronary plaques using non-contrast T1w imaging could facilitate the prediction of pMI after elective PCI. Previous reports have shown that characterizing coronary HIPs with non-contrast T1w imaging is a promising noninvasive method and a novel biomarker for identifying high-risk coronary plaques without any radiation or iodinated contrast exposure [4–7]. Currently, coronary plaques on T1w imaging are quantified using the signal intensity ratio between plaque and a reference point (i.e., nearby myocardium). This value, 2D-PMR, characterizes plaques without taking into account the concept of plaque volume. Matsumoto et al. reported a relationship between qualitative coronary PMR morphology (e.g., intrawall or intraluminal high-intensity signals) visualized with non-contrast T1w imaging and their clinical severity [19]. In addition, since larger coronary plaque volume was associated with future cardiovascular events [8, 9, 20], we have developed a novel technique for quantitative 3D plaque analysis with non-contrast T1w imaging (Figs. 1 and 2). Indeed, as shown in Table 3, the present 3D coronary plaque assessment produces a more sensitive predictor of pMI after elective PCI than current 2D-PMR and indices derived from IVUS or coronary angiography. It may also be a better predictor of pMI than CTA-derived factors (Additional file 1: Tables S1–S2), because CMR was reported to be superior to CTA in terms of characterizing noncalcified, atherosclerotic plaques in an experimental rabbit atherosclerotic model [21], and current 3D plaque assessment may involve both plaque characteristics and their volume. As shown in Figure 5, the prevalence of pMI was higher in patients with 3Di-PMR ≥ 51 PMR*mm^3^ than in those with 3Di-PMR < 51 PMR*mm^3^, irrespective of their 2D-PMR value. Additional file 1: Table S3 also showed that patients with higher 3Di-PMR had positive vessel remodeling compared with patients with lower 3Di-PMR, and patients with higher 2D-PMR had longer attenuation length compared with patients with lower 2D-PMR. Because coronary plaque volume and composition play an important role in pMI after elective PCI, the present 3D evaluation using non-contrast T1w imaging for coronary atherosclerosis may improve the accuracy of predicting cardiac events. To decrease the incidence of pMI and improve clinical outcomes, this noninvasive preoperative evaluation before scheduled PCI might facilitate risk stratification for subgroups at high risk for pMI, who might then benefit from intensive pharmacological approaches (e.g., statins and antiplatelet therapy) and usage of filter devices during elective PCI procedures [22–25].

As shown in Figure 3 and Table 2, total plaque volume and lipid-rich plaque as assessed by IVUS were significantly correlated with 3Di-PMR. In carotid atherosclerosis, HIP atherosclerotic lesions detected by non-contrast T1w imaging consist of complex atheromas with large necrotic cores and intraplaque hemorrhage [26, 27]. Our previous case report showed that coronary emboli during PCI, detected as a coronary HIP on non-contrast T1w imaging before PCI, is composed of a large necrotic core with cholesterol crystals and thrombus [10]. Additionally, a histological analysis using formalin-fixed post-mortem human hearts, which were imaged at 1.5 T with T1w imaging with fat suppression, showed that the majority of coronary HIPs on T1w imaging was reflect intraplaque hemorrhage [28]. Thus, coronary HIPs detected on T1w imaging could include complex atheromas with intraplaque hemorrhage. The precise relationship between plaque burden with intraplaque hemorrhage and pMI during PCI remains unclear. Further studies are needed to clarify the relationship among MRI findings, associated histological and molecular characteristics, and clinical events in coronary atherosclerosis.

### Limitations

Our study has several limitations. The number of patients was small and there might have been selection bias related to how patients were chosen for CMR. Second, the algorithm for 3D plaque evaluation requires discussion. The Step 1 algorithm, which is based on automated 3D region growing for a coronary plaque with higher signal intensity than nearby myocardium, allowed for 3D quantification of coronary plaques in 128 of 141 patients (91%). The other 13 patients required additional steps. In particular, the Step 3 algorithm required an increment of the lower segmentation threshold for plaque segmentation until there was no longer expansion beyond the vessel boundaries. These additional steps could hamper this assessment in more complex lesions and have little impact on predicting clinical outcomes. However, there is currently no method to quantify plaque morphology on T1w imaging. Thus, further studies to develop plugin software or other algorithms for plaque segmentation are needed for 3D plaque quantification with CMR in coronary atherosclerosis. Third, we did not perform systemic analyses to compare the findings between coronary CMR and CTA, which could also predict pMI.

## Conclusion

Three-dimensional assessment of coronary plaques reflecting both plaque volume and composition facilitates more accurate risk stratification and prediction of pMI after elective PCI in stable CAD patients.

## Supplementary information

**Additional file 1: **Supplemental Methods, Tables, and Figures. **Table S1.** Lesion Characteristics on Coronary CTA. **Table S2.** Receiver Operating Characteristic Analysis Demonstrating the Prediction of pMI using 3Di-PMR and Coronary CTA Variables. **Table S3.** Lesion Characteristics Categorized by 2D-PMR and 3Di-PMR Cutoffs. **Figure S1.** Step 1 algorithm for 3-dimensional assessment of coronary plaque (3D Region-growing technique). **Figure S2-a.** Step 2 algorithm. **b.** vessel diameter and plaque segmentation. **Figure S3.** Step 3 algorithm. **Figure S4.** Troponin T levels before and after PCI in (A) patients without pMI and (B) those with pMI. (C) Relationship between 2D-PMR and 3Di-PMR on T1w imaging. (D, E) Incidence of pMI (A) and slow flow by 3Di-PMR cutoff value.

**Additional file 2: Step 1, Video S1.**

**Additional file 3: Step 2, Video S2.**

**Additional file 4: Step 3, Video S3.**

## Data Availability

The datasets used and/or analyzed during the current study are available from the corresponding author on reasonable request.

## Abbreviations

2D: 2-dimensional
3D: 3-dimensional
3Di: 3-dimensional integral
CAD: Coronary artery disease
CI: Confidence interval
CMR: Cardiovascular magnetic resonance
CTA: Coronary computed tomography angiography
ECG: Electrocardiogram
HIP: High intensity plaque
hs-TnT: High-sensitivity troponin T
IQR: Interquartile range
IVUS: Intravascular ultrasound
OR: Odds ratio
PCI: Percutaneous coronary intervention
pMI: Periprocedural myocardial injury
PMR: Plaque-to-myocardial signal intensity ratio
ROI: Region of interest
T1w: T1-weighted
TIMI: Thrombolysis In Myocardial Infarction
