# Correction to: Three-dimensional assessment of coronary high-intensity plaques with T1-weighted cardiovascular magnetic resonance imaging to predict periprocedural myocardial injury after elective percutaneous coronary intervention

**DOI:** 10.1186/s12968-020-00620-4

**Published:** 2020-04-27

**Authors:** Hayato Hosoda, Yasuhide Asaumi, Teruo Noguchi, Yoshiaki Morita, Yu Kataoka, Fumiyuki Otsuka, Kazuhiro Nakao, Masashi Fujino, Toshiyuki Nagai, Michikazu Nakai, Kunihiro Nishimura, Atsushi Kono, Yoshiaki Komori, Tomoya Hoshi, Akira Sato, Tomohiro Kawasaki, Chisato Izumi, Kengo Kusano, Tetsuya Fukuda, Satoshi Yasuda

**Affiliations:** 1 Department of Cardiovascular Medicine, National Cerebral and Cardiovascular Center, 6-1 Kishibe-Shimmachi, Suita, 564-8565 Japan; 2 Department of Advanced Cardiovascular Medicine, Graduate School of Medical Sciences, Kumamoto University, Kumamoto, Japan; 3 Department of Radiology, National Cerebral and Cardiovascular Center, Suita, Japan; 4 Department of Preventative Cardiology, National Cerebral and Cardiovascular Center, Suita, Japan; 5 Department of Research and Collaboration, Siemens Japan KK, Tokyo, Japan; 6 Department of Cardiovascular Medicine, University of Tsukuba, Tsukuba, Japan; 7 Cardiovascular Center, Shin-Koga Hospital, Kurume, Japan

**Correction to: J Cardiovasc Magn Reson**

**https://doi.org/10.1186/s12968-019-0588-6**

In the original publication of this article [1] the wording of ‘3Di-PMR’ was different between the text and figures. Figures 1, 3, 4 and 5 contained the old wording ‘3D-PMRI’. In this correction article the updated figures are published.

**Fig. 1:**
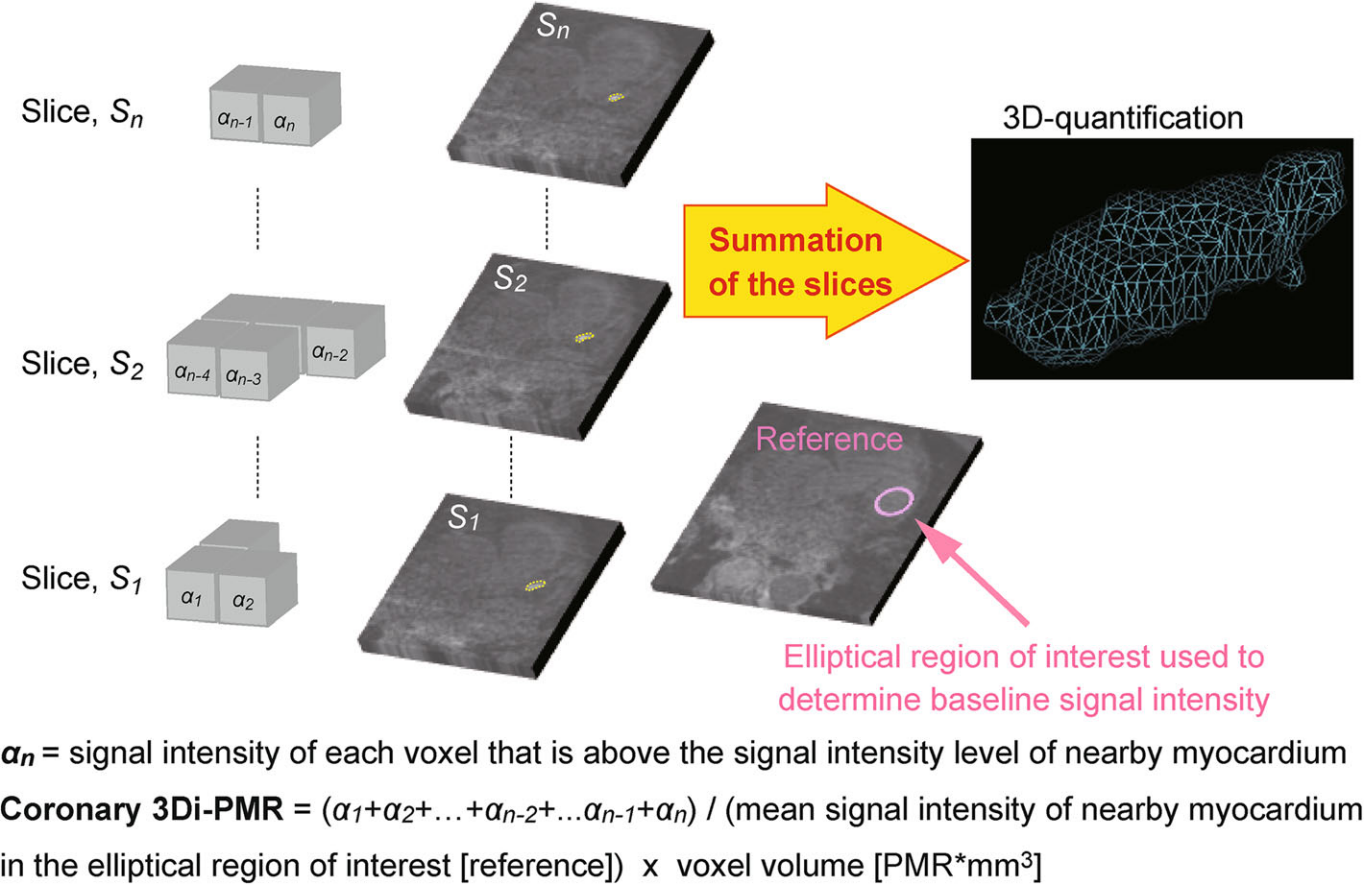
Principle behind 3-dimensional (3D) plaque assessment on T1-weighted imaging. Gray cubes represent voxels. α_n_ represents the signal intensity of each voxel with higher signal intensity than that of nearby myocardium. Entire voxels of a coronary plaque that are above the signal intensity of nearby myocardium (plaque-to-myocardial signal intensity ratio > 1.0) were segmented within contiguous slices (surrounded by yellow dotted lines) to calculate the integral of signal intensity and voxel volume

**Fig. 3:**
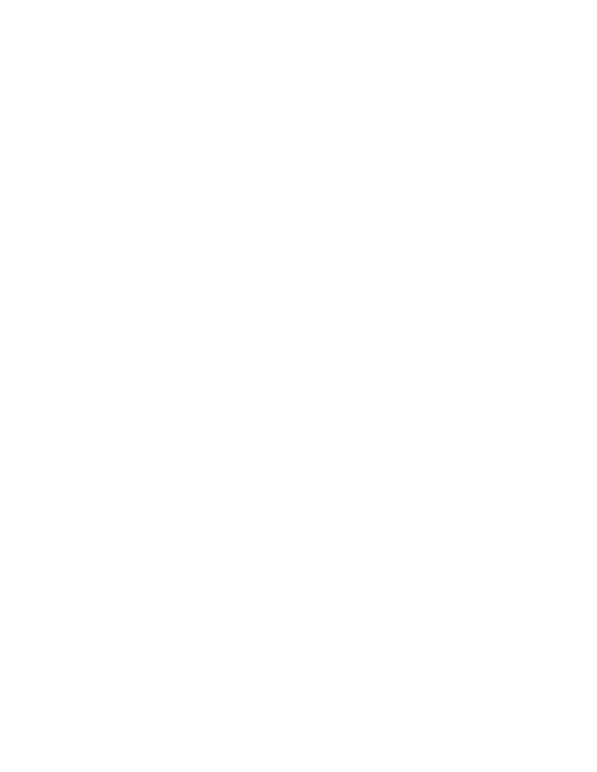
Correlation between 3D integral (3Di)-plaque to myocardial signal intensity ratio (PMR) and plaque characteristics based on integrated backscatter intravascular ultrasound. Correlation between 3Di-PMR and total plaque volume (**a**), lipid plaque volume (**b**), fibrous plaque volume (**c**), and calcified plaque volume (**d**) are shown

**Fig. 4:**
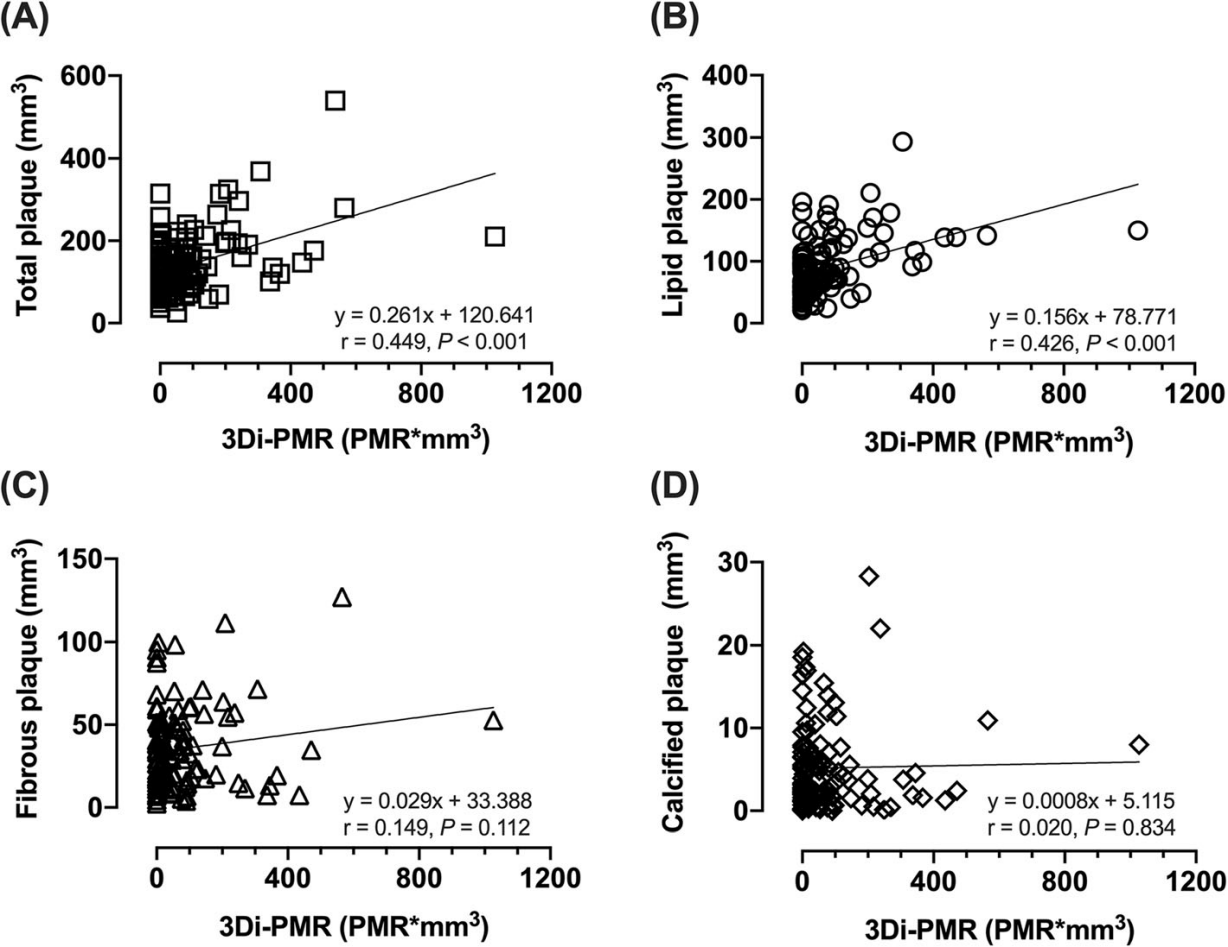
Representative 2-dimensional and 3-dimensional plaque assessment on T1-weighted imaging. Coronary plaques with 2D^low^3D^high^ in the proximal right coronary artery (2D-PMR, 1.14; 3Di-PMR, 237 PMR*mm^3^; Patient A: a–e), 2D^high^3D^low^ in the proximal left anterior descending artery (LAD) (2D-PMR, 1.50; 3Di-PMR, 43 PMR*mm^3^; Patient B: f–j), and 2D^high^3D^high^ in the proximal LAD (2D-PMR, 1.96; 3Di-PMR, 344 PMR*mm^3^; Patient C: k–o). Computed tomography angiography (CTA) images (a, f, k), and axial images (b, g, l), sagittal images (c, h, m), color maps (d, I, n), and 3D region of interests (3D plaque: e, j, n) on T1w images are shown. Yellow circles indicate percutaneous coronary intervention target lesion sites on CTA. Yellow arrows indicate lesions on T1w imaging corresponding to a lesion on angiography that underwent intervention

**Fig. 5:**
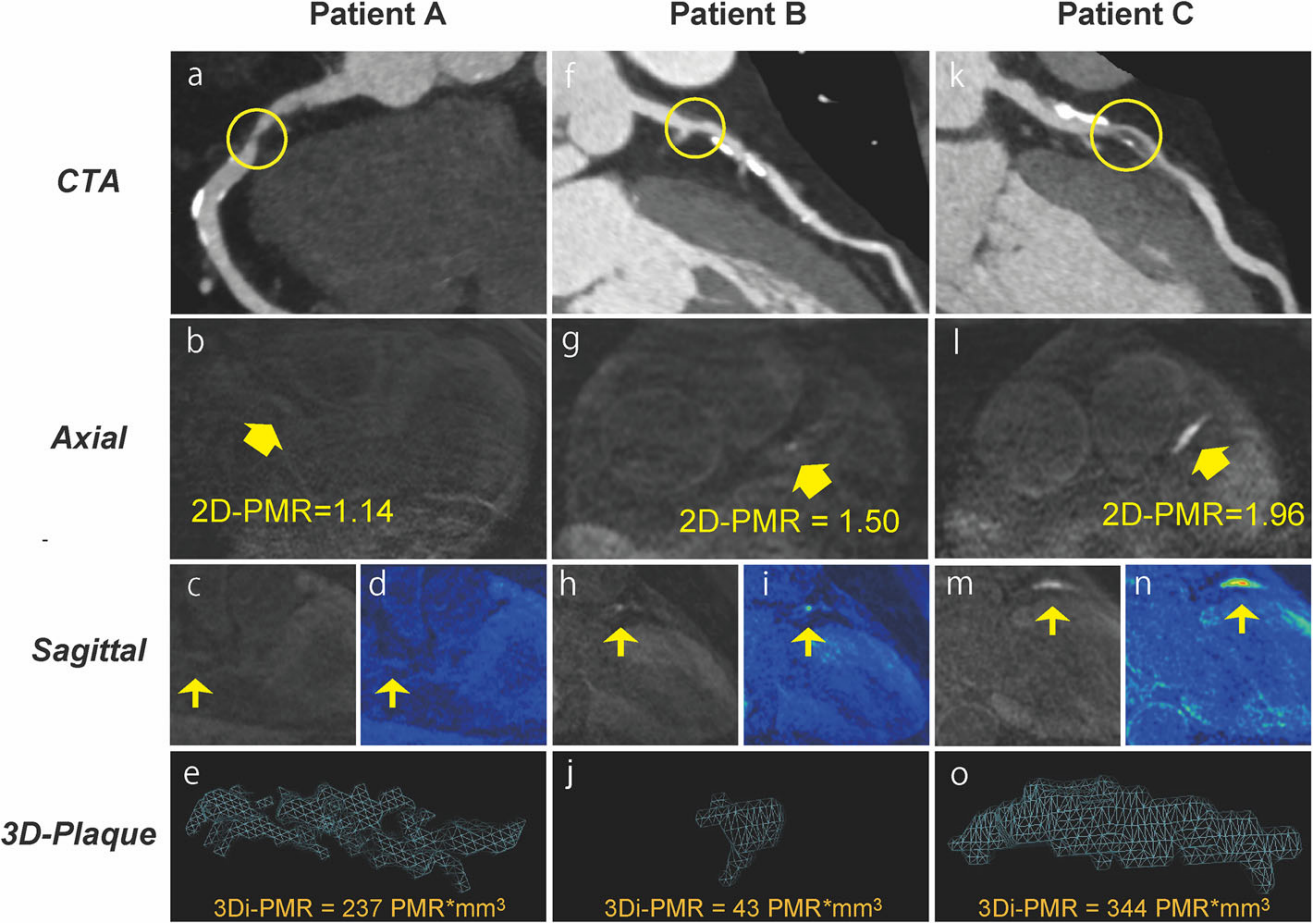
Incidence of periprocedural myocardial injury (pMI) based on 3Di-PMR and 2D-PMR cutoff values. The red and blue bars represent patients with 3Di-PMR ≥ 51 PMR*mm^3^ and < 51 PMR*mm^3^, respectively. *P* < 0.001 based on the chi-squared test. * *P* = 0.006 vs. 2D^high^3D^low^ group. ^†^*P* < 0.001 vs. 2D^low^3D^low^ group, and *P* = 0.003 vs. 2D^high^3D^low^ group
