# Supplementary material for: Three-dimensional assessment of coronary high-intensity plaques with T1-weighted cardiovascular magnetic resonance imaging to predict periprocedural myocardial injury after elective percutaneous coronary intervention
Source: J Cardiovasc Magn Reson. 2020 Jan 16;22:5. doi: 10.1186/s12968-019-0588-6 (PMC6964021; doi:10.1186/s12968-019-0588-6)
Supplement: Supplementary file 1 — Additional file 1: Supplemental Methods, Tables, and Figures. Table S1. Lesion Characteristics on Coronary CTA. Table S2. Receiver Operating Characteristic Analysis Demonstrating the Prediction of pMI using 3Di-PMR and Coronary CTA Variables. Table S3. Lesion Characteristics Categorized by 2D-PMR and 3Di-PMR Cutoffs. Figure S1. Step 1 algorithm for 3-dimensional assessment of coronary plaque (3D Region-growing technique). Figure S2-a. Step 2 algorithm. b. vessel diameter and plaque segmentation. Figure S3. Step 3 algorithm. Figure S4. Troponin T levels before and after PCI in (A) patients without pMI and (B) those with pMI. (C) Relationship between 2D-PMR and 3Di-PMR on T1w imaging. (D, E) Incidence of pMI (A) and slow flow by 3Di-PMR cutoff value. [file 12968_2019_588_MOESM1_ESM.docx]

**Supplementary Materials**

**Three-dimensional assessment of coronary high-intensity plaques with T1-weighted cardiovascular magnetic resonance imaging to predict periprocedural myocardial injury after elective percutaneous coronary intervention.**

Hayato Hosoda MD^1,2^, Yasuhide Asaumi MD PhD^1^, Teruo Noguchi MD PhD^1^, Yoshiaki Morita MD PhD^3^, Yu Kataoka MD PhD^1,2^, Fumiyuki Otsuka MD PhD^1^, Kazuhiro Nakao MD PhD^1^, Masashi Fujino MD PhD^1^, Toshiyuki Nagai MD PhD^1^, Michikazu Nakai PhD^4^, Kunihiro Nishimura MD PhD^4^, Atsushi Kono MD PhD^3^ Yoshiaki Komori MSc^5^, Tomoya Hoshi MD PhD^6^, Akira Sato MD PhD^6^, Tomohiro Kawasaki MD^7^, Chisato Izumi MD PhD^1^, Kengo Kusano MD PhD^1^, Tetsuya Fukuda MD PhD^3^, and Satoshi Yasuda MD PhD^1,2^

1. Department of Cardiovascular Medicine, National Cerebral and Cardiovascular Center, Suita, Japan

2. Department of Advanced Cardiovascular Medicine, Graduate School of Medical Sciences, Kumamoto University, Kumamoto, Japan

3. Department of Radiology, National Cerebral and Cardiovascular Center, Suita, Japan

4. Department of Preventative Cardiology, National Cerebral and Cardiovascular Center, Suita, Japan

5. Department of Research and Collaboration, Siemens Japan KK, Tokyo, Japan

6. Department of Cardiovascular Medicine, University of Tsukuba, Tsukuba, Japan

7. Cardiovascular Center, Shin-Koga Hospital, Kurume, Japan

**Address for correspondence:**

Yasuhide Asaumi MD, PhD

Department of Cardiovascular Medicine

National Cerebral and Cardiovascular Center

6-1 Kishibe-Shimmachi, Suita, 564-8565, Japan

Tel: +81-6-6170-1070

Fax: +81-6-6170-1782

E-mail: [asaumiya@ncvc.go.jp](mailto:asaumi.yasuhide.hp@ncvc.go.jp)

**Supplemental Methods**

**Intravascular ultrasound image analysis**

Intravascular ultrasound (IVUS) images were obtained during PCI and analyzed in 126 patients. IVUS was performed using a commercially available IVUS catheter (View It; Terumo, Tokyo, Japan) with 0.5-mm/sec auto-pullback. Quantitative and qualitative analyses were performed in a blinded manner. Positive remodeling was defined as a remodeling index of >1.05. Ultrasound attenuation was defined as backward signal attenuation of ≥180° behind a plaque, with no evidence of dense calcium deposition, and with a longitudinal attenuation length of ≥5 mm.^16^ For tissue characterization, IVUS data were analyzed based on previous data used to define a range of integrated backscatter values; the manufacturer’s default setting was used. Coronary plaques were classified into three categories: lipid pool (blue), fibrosis (green/yellow), and calcification (red).^1^

**Coronary Computed Tomography Angiography Protocol**

Coronary images were acquired with 128 × 0.6-mm slice collimation, 280-ms gantry rotation time, 120-kV tube voltage, and 280-mAs quality reference current-time product using a dual-source CT scanner (SOMATOM Definition Flash; Siemens Healthcare, Erlangen, Germany). The images were reconstructed with 0.6-mm slice thickness in 0.3-mm increments with a medium smooth convolution kernel. Image analysis was performed using a commercially available workstation (Ziostation2; Ziosoft, Tokyo, Japan). Multiplanar reconstruction and cross-sectional images of the lesion were used to measure the cross-sectional area (CSA) of the external elastic membrane (EEM) of vessels. The remodeling index was defined as the EEM CSA of the target lesion divided by the average EEM CSA of the proximal and distal references. Plaque CT density was assessed at ≥3 points and averaged. Spotty calcification was defined as calcifications <3 mm in size on focal multiplanar reconstruction and cross-sectional images.^2, 3^

**Table S1. Lesion Characteristics on Coronary CTA**

|  | **n=57** |
| --- | --- |
| Age, years | 68 (61–73) |
| Male | 47 (82) |
| pMI | 19 (33) |
| Slow flow | 8 (14) |
| 2D-PMR | 1.17 (1.02–1.63) |
| 3Di-PMR, PMR*mm^3^ | 87 (19–261) |
| **CTA measurements** |  |
| Lesion EEM CSA, mm^2^ | 12 (7.9–16.7) |
| Lesion MLA, mm^2^ | 1.75 (1.28–2.4) |
| Lesion length, mm | 23 (18–30) |
| CT attenuation value, HU | 60 (28–88) |
| LAP | 22 (39) |
| Remodeling index | 1.22 (0.82–1.46) |
| Positive remodeling | 33 (58) |
| Spotty calcification | 26 (46) |

Values are medians (interquartile range) or n (%).

2D-PMR, 2-dimensional plaque-to-myocardial signal intensity ratio; 3Di-PMR, 3-dimensional integral of the plaque-to-myocardium signal intensity ratio; CSA, cross-sectional area; CTA, computed tomography angiography; EEM, external elastic membrane; LAP, low-attenuation plaque; MLA, minimum lesion area; pMI, periprocedural myocardial injury.

Positive remodeling was defined by a remodeling index >1.05.^1^

LAP was defined by a CT attenuation value <50 Hounsfield units.^2^

**Table S2. Receiver Operating Characteristic Analysis Demonstrating the Prediction of pMI using 3Di-PMR and Coronary CTA Variables.**

| Variable | AUC (95% CI) | *P* Value |
| --- | --- | --- |
| 3Di-PMR | 0.777 (0.644–0.910) | Referent |
| Remodeling index | 0.618 (0.461–0.774) | 0.051 |
| CT value | 0.609 (0.458–0.760) | 0.051 |
| Positive remodeling | 0.579 (0.445–0.713) | 0.007 |
| LAP | 0.566 (0.427–0.704) | 0.007 |
| Spotty calcification | 0.526 (0.367–0.666) | 0.022 |
| LAP + positive remodeling | 0.651 (0.501–0.801) | 0.068 |

3Di-PMR, 3-dimensional integral of the plaque-to-myocardium signal intensity ratio; CI, confidence interval; CT, computed tomography; CTA, computed tomography angiography; LAP, low-attenuation plaque; pMI, periprocedural myocardial injury; RI, remodeling index.

Positive remodeling was defined by a remodeling index >1.05.^1^

LAP was defined by a CT attenuation value <50 Hounsfield units.^2^

**Table S3. Lesion Characteristics Categorized by 2D-PMR and 3Di-PMR Cutoffs**

|  | 2D-PMR < 1.4 (n=93) | |  | 2D-PMR ≥ 1.4 (n=48) | | *P* Value |
| --- | --- | --- | --- | --- | --- | --- |
|  | 3Di-PMR < 51 PMR*mm^3^  (n=79) | 3Di-PMR ≥ 51 PMR*mm^3^  (n=14) |  | 3Di-PMR < 51 PMR*mm^3^  (n=6) | 3Di-PMR ≥ 51 PMR*mm^3^  (n=42) |  |
| Type B2/C lesion | 41 (52) | 8 (57) |  | 5 (83) | 27 (64) | 0.328 |
| Calcification | 14 (18) | 2 (13) |  | 3 (50) | 5 (12) | 0.139 |
| Side branch | 47 (60) | 9 (60) |  | 6 (100) | 24 (57) | 0.102 |
| ***Cross-sectional IVUS analysis*** | | | | | | |
| **Grayscale IVUS parameters** | (n=68) | (n=15) |  | (n=5) | (n=38) |  |
| Lesion EEM CSA, mm^2^ | 8.4 (6.0–11.4) | 12.8 (5.8–14.3) |  | 7.0 (6.4–9.3)† | 12.9 (9.6–15.9)* | <0.001 |
| Lesion lumen CSA, mm^2^ | 1.3 (0.98–1.98) | 1.03 (0.81–1.22) |  | 1.03 (0.69–1.30) | 1.36 (0.87–2.05) | 0.039 |
| Lesion P+M CSA mm^2^ | 6.6 (4.9–9.5) | 11.3 (4.5–13.3) |  | 6.0 (5.1–8.6) | 11.2 (8.3–13.6)* | <0.001 |
| Plaque burden, % | 82 (77–86) | 90 (84–94)* |  | 85 (80–93) | 89 (85–92)* | <0.001 |
| Remodeling index | 0.88 (0.78–1.03) | 1.01 (0.79–1.17) |  | 1.02 (0.88–1.08) | 1.11 (0.93–1.22)* | <0.001 |
| Positive remodeling | 16 (24) | 7 (47) |  | 2 (40) | 20 (53) | 0.019 |
| Attenuation length | 0 (0–0.6) | 1 (0–3.5)† |  | 3.4 (1.3–5.9)* | 4.3 (2.3–7.3)* | <0.001 |
| Ultrasound attenuation | 4 (6) | 0 (0) |  | 2 (40) | 18 (47) | <0.001 |
| Intracoronary thrombus | 2 (3) | 1 (8) |  | 0 (0) | 8 (21) | 0.076 |
| **IB-IVUS parameters** |  |  |  |  |  |  |
| Lipid area, % | 68 (54–80) | 75 (63–87) |  | 75 (64–87) | 82 (72–87)* | 0.007 |
| Fibrous area, % | 24 (17–39) | 24 (14–32) |  | 23 (12–35) | 16 (12–24)* | 0.010 |
| Calcified area, % | 3.8 (1.1–7.6) | 1.6 (0.5–3.1) |  | 1.6 (0.3–2.7) | 2.3 (0.4–3.9) | 0.047 |
| ***Volumetric IVUS analysis*** | | | | | | |
| **Grayscale IVUS parameters** | | | | | | |
| EEM volume, mm^3^ | 174 (135–247) | 263 (118–338) |  | 136 (132–207) | 244 (176–332)* | 0.008 |
| Lumen volume, mm^3^ | 61 (48–101) | 79 (46–104) |  | 62 (40–67) | 75 (58–113) | 0.336 |
| Total plaque volume, mm^3^ | 106 (81–140) | 160 (72–208) |  | 103 (78–140) | 152 (120–211)* | 0.001 |
| Plaque burden, % | 60 (54–67) | 65 (61–68) |  | 66 (59–72) | 68 (62–75)* | 0.003 |
| **IB-IVUS parameters** |  |  |  |  |  |  |
| Lipid volume, % | 66 (54–77) | 71 (62–80) |  | 73 (59-83) | 75 (62-84)* | 0.154 |
| Fibrous volume, % | 29 (20–41) | 28 (17–34) |  | 26 (15–39) | 22 (14–33) | 0.204 |
| Calcified volume, % | 3.7 (1.6–7.9) | 2.2 (0.8–4.4) |  | 2.0 (0.6–3.2) | 2.5 (0.8–4.3) | 0.093 |

Values are medians (interquartile range) or n (%).

2D-PMR, 2-dimensional plaque-to-myocardium signal intensity ratio; 3Di-PMR, 3-dimensional integral of the plaque-to-myocardium signal intensity ratio; CSA, cross-sectional area; EEM, external elastic membrane; IB-IVUS, integrated backscatter intravascular ultrasound; IVUS, intracoronary ultrasound; P+M, plaque plus media**.**

Multiple comparisons were analyzed using the Kruskal-Wallis test followed by the Steel-Dwass test for post hoc analysis.

* *P*<0.01 compared with patients with 2D-PMR < 1.4 + 3Di-PMR < 51 PMR*mm^2^.

^†^ *P*<0.05 compared with patients with 2D-PMR ≥ 1.4 + 3Di-PMR ≥ 51 PMR* mm^2^.

**Figure S1. Step 1 Algorithm for 3-Dimensional Assessment of Coronary Plaque (3D Region-growing Technique)**

**
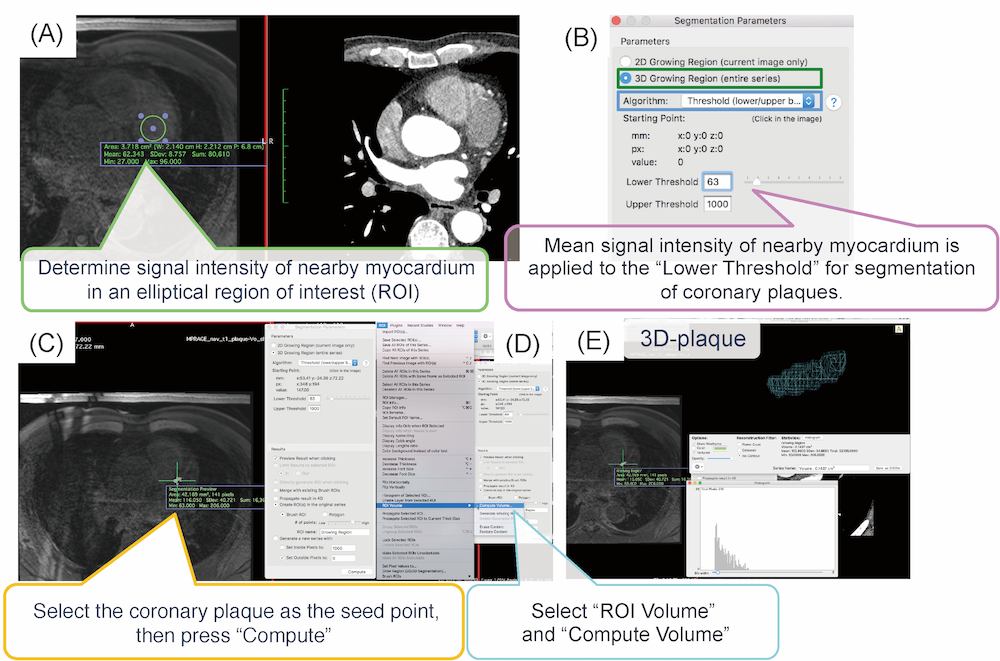
**

(A) Determine the signal intensity of nearby myocardium in an elliptical region of interest (ROI) that is set as the lower threshold of the coronary plaque signal intensity.

(B) Segmentation parameters are determined using the “3D Growing Region” (green box). Thereafter, the mean signal intensity of nearby myocardium is applied to the “Lower Threshold” for the segmentation of coronary plaques.

(C) Select the coronary plaque as the seed point and press “Compute.”

(D) Select “ROI Volume” and “Compute Volume.”

(E) Final 3-dimensional plaque segmentation.

See Online Video 1.

**Figure S2-a. Step 2 Algorithm**

**
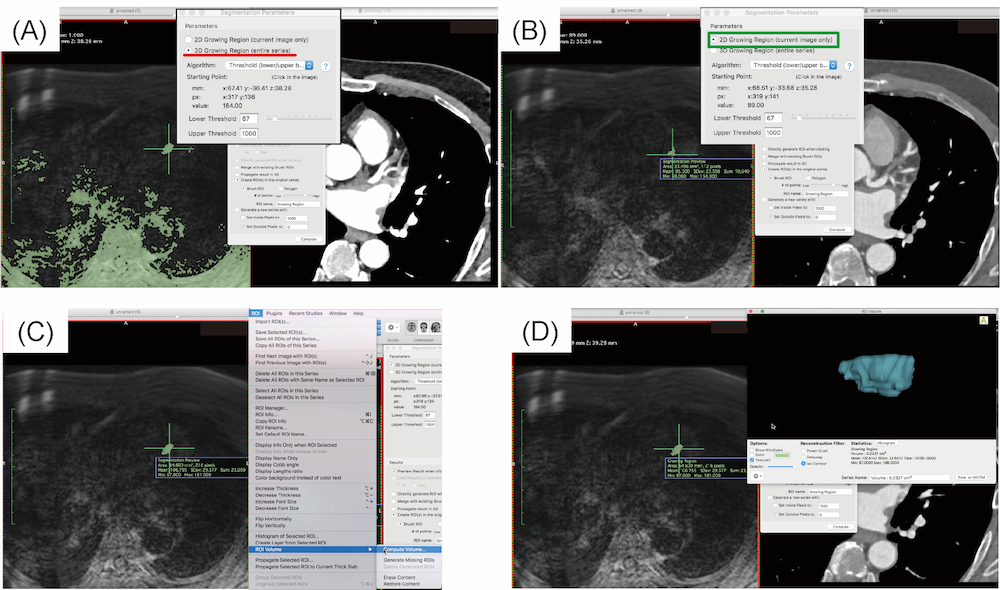
**

(A–B) If the ROI expands beyond the boundaries of the vessel when using “3D Growing Region,” select “2D Growing Region” (green box in B) instead of “3D Growing Region” (red underline in A). For each slice, after “2D Growing Region” has been selected, press “Compute.”

(C) After automatic 2D segmentation of a coronary plaque has occurred above the PMR threshold without expansion beyond the boundaries of the vessel and in 5 contiguous slices, select “Compute Volume.”

(D) Final 3-dimensional plaque segmentation.

See Online Video 2.

**Figure S2-b. Vessel Diameter and Plaque Segmentation**

**
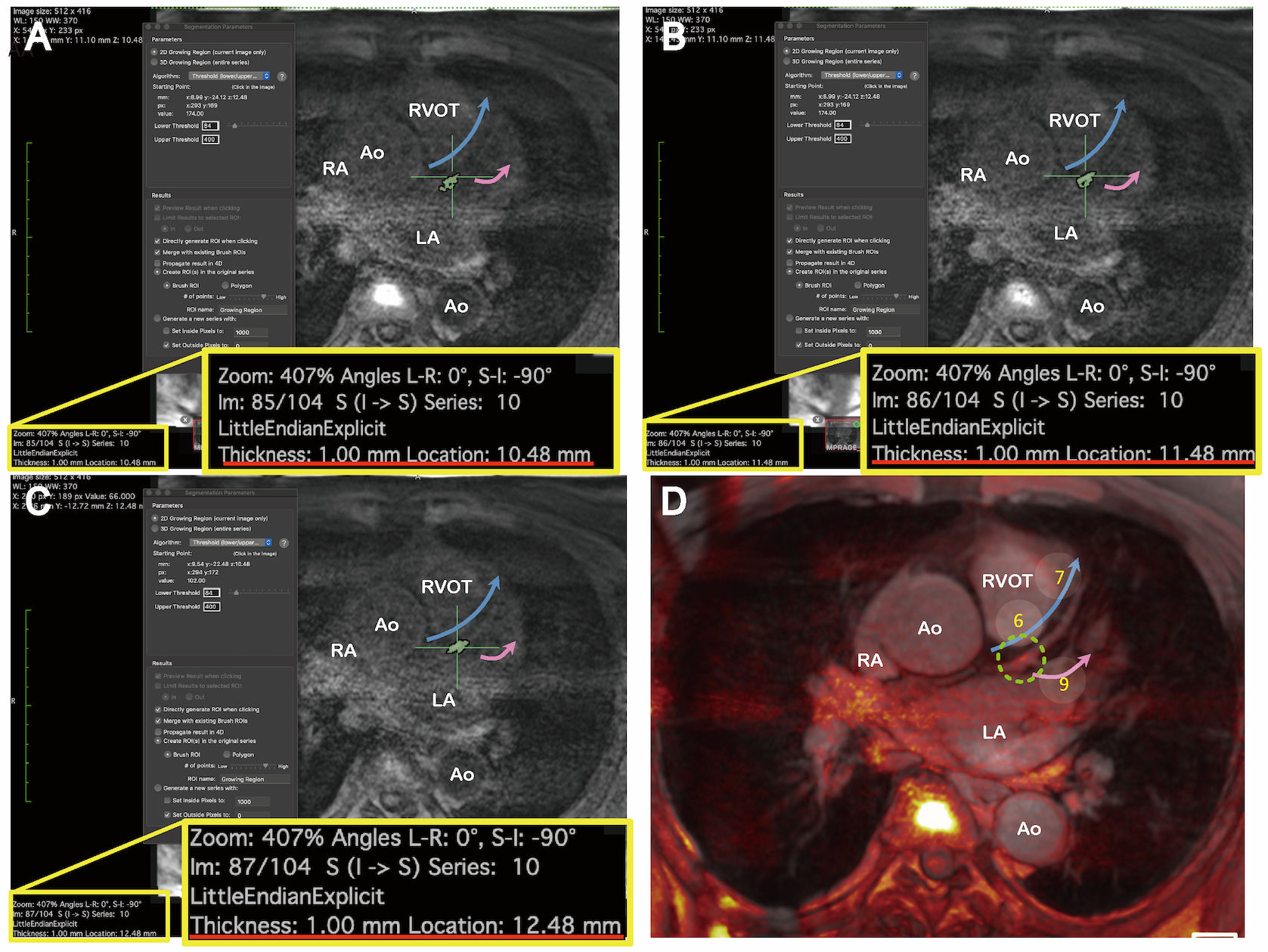
**

(A-C) Three consecutive 1-mm slices on T1WI. The green cross shows 2-dimensional plaque segmentation above the signal intensity of nearby myocardium.

The curved blue arrow represents the proximal LAD (segments 6 and 7). The curved pink arrow represents the proximal diagonal branch (segment 9).

(D) Merged image comprising MRA and T1WI.

The orange-colored segment within the dotted green circle represents plaque with a higher signal intensity than nearby myocardium. The curved blue arrow represents the proximal LAD (segments 6 and 7: circled numbers). The curved pink arrow represents the proximal diagonal branch (segment 9: circled number).

Ao, Aorta; LA, Left atrium; RA, Right atrium; RVOT, right ventricular outflow tract.

**Figure S3. Step 3 Algorithm**

**
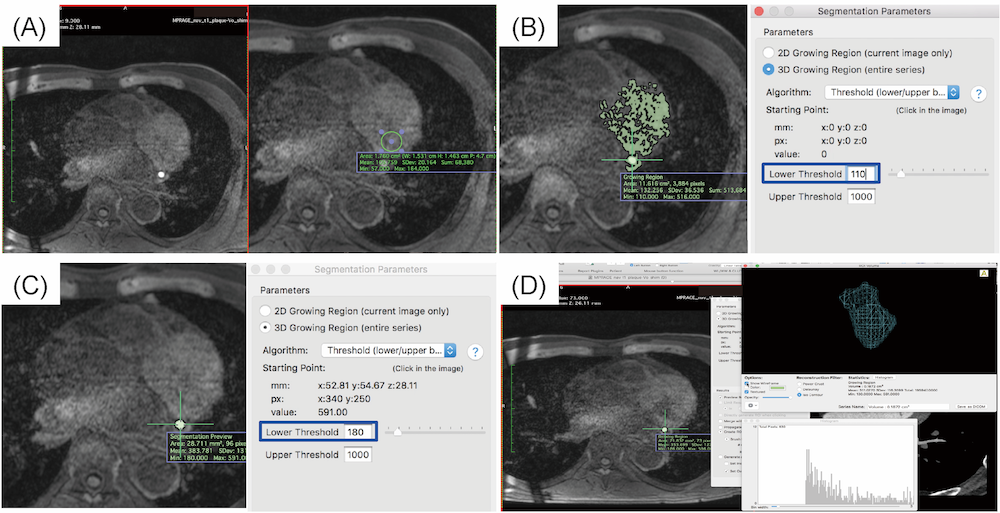
**

(A–C) Three-dimensional segmentation of coronary plaques in segments 2, 11, and 13 (running perpendicular to a given axial section) could not be performed with Step 1. The lower segmentation threshold was increased from the signal intensity of the nearby myocardium in increments of 5 units until there was no longer expansion beyond the vessel boundaries (blue boxes in B and C).

(D) Final 3-dimensional plaque segmentation.

See Online Video 3.

**Figure S4.**

**
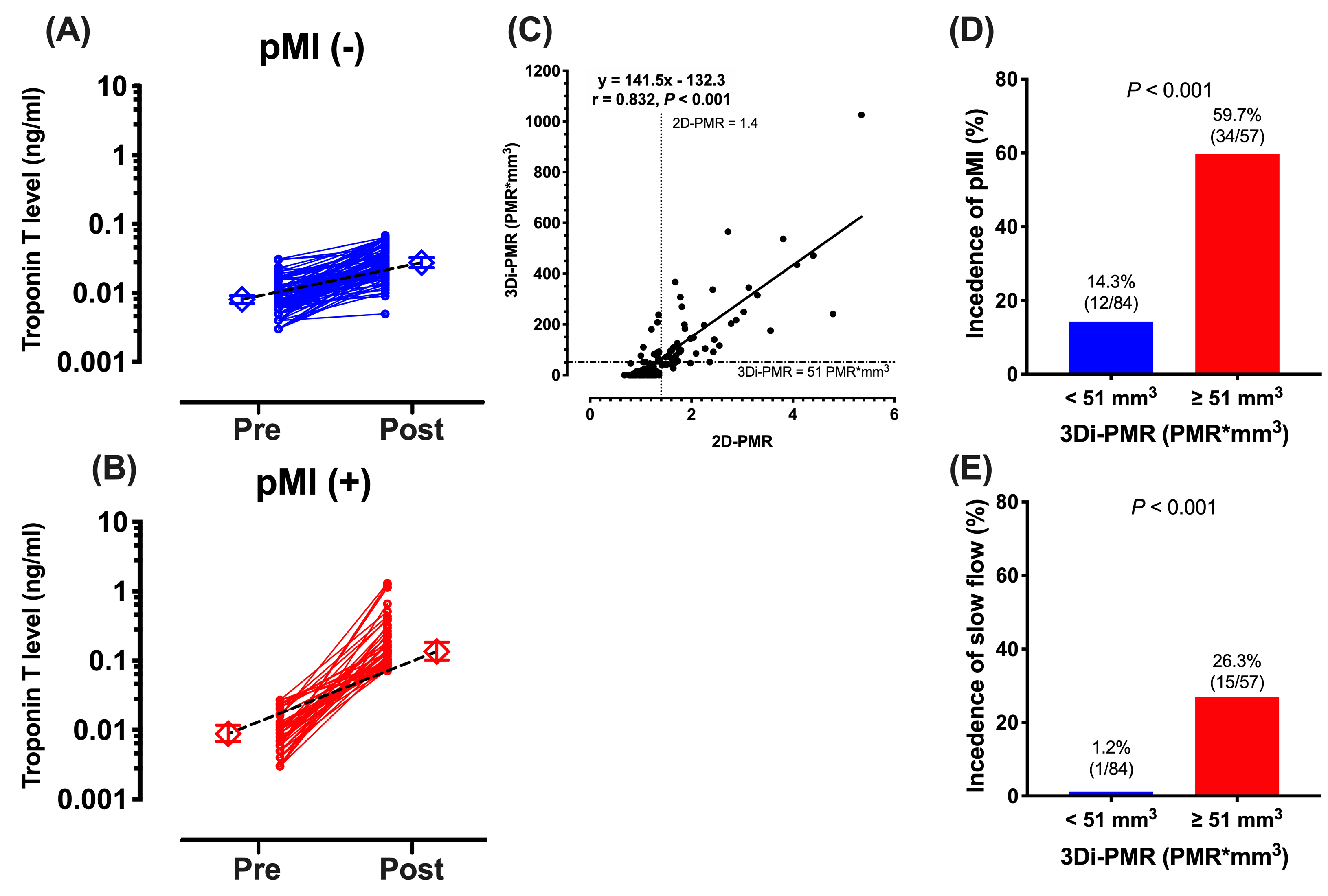
**

Troponin T levels before and after PCI in (A) patients without pMI and (B) those with pMI. Circles (red or blue) represent each high-sensitivity troponin T level and open diamonds and error bars (red or blue) at pre- and post-PCI represent their medians and 95% confidence intervals in patients with or without pMI, respectively.

(C) Relationship between 2D-PMR and 3Di-PMR on T1WI. 3Di-PMR is strongly and positively correlated with 2D-PMR.

(D, E) Incidence of pMI (A) and slow flow by 3Di-PMR cutoff value (B) The red and blue bars represent the incidence of slow flow and pMI, respectively, based on the 3Di-PMR cutoff value of 51 PMR*mm3.

2D-PMR, 2-dimensional plaque-to-myocardial signal intensity ratio; 3Di-PMR, 3-dimensional integral of the plaque-to-myocardium signal intensity ratio; pMI, periprocedural myocardial injury.

**Reference**

1. Hoshi T, Sato A, Akiyama D, Hiraya D, Sakai S, Shindo M, Mori K, Minami M, Aonuma K. Coronary high-intensity plaque on T1-weighted magnetic resonance imaging and its association with myocardial injury after percutaneous coronary intervention. Eur Heart J 2015;**36**(29):1913-22.

2. Watabe H, Sato A, Akiyama D, Kakefuda Y, Adachi T, Ojima E, Hoshi T, Murakoshi N, Ishizu T, Seo Y, Aonuma K. Impact of coronary plaque composition on cardiac troponin elevation after percutaneous coronary intervention in stable angina pectoris: a computed tomography analysis. J Am Coll Cardiol 2012;**59**(21):1881-8.

3. Uetani T, Amano T, Kunimura A, Kumagai S, Ando H, Yokoi K, Yoshida T, Kato B, Kato M, Marui N, Nanki M, Matsubara T, Ishii H, Izawa H, Murohara T. The association between plaque characterization by CT angiography and post-procedural myocardial infarction in patients with elective stent implantation. JACC Cardiovasc Imaging 2010;**3**(1):19-28.
